# Thermal Assisted Oxygen Annealing for High Efficiency Planar CH_3_NH_3_PbI_3_ Perovskite Solar Cells

**DOI:** 10.1038/srep06752

**Published:** 2014-10-24

**Authors:** Zhiwei Ren, Annie Ng, Qian Shen, Huseyin Cem Gokkaya, Jingchuan Wang, Lijun Yang, Wai-Kin Yiu, Gongxun Bai, Aleksandra B. Djurišić, Wallace Woon-fong Leung, Jianhua Hao, Wai Kin Chan, Charles Surya

**Affiliations:** 1 Department of Electronic and Information Engineering, The Hong Kong Polytechnic University, Hong Kong SAR; 2 Department of Mechanical Engineering, The Hong Kong Polytechnic University, Hong Kong SAR; 3 Department of Physics, The University of Hong Kong, Pokfulam, Hong Kong SAR; 4 Department of Applied Physics, The Hong Kong Polytechnic University, Hong Kong SAR; 5 Department of Chemistry, The University of Hong Kong, Pokfulam, Hong Kong SAR

## Abstract

We report investigations on the influences of post-deposition treatments on the performance of solution-processed methylammonium lead triiodide (CH_3_NH_3_PbI_3_)-based planar solar cells. The prepared films were stored in pure N_2_ at room temperature or annealed in pure O_2_ at room temperature, 45°C, 65°C and 85°C for 12 hours prior to the deposition of the metal electrodes. It is found that annealing in O_2_ leads to substantial increase in the power conversion efficiencies (PCEs) of the devices. Furthermore, strong dependence on the annealing temperature for the PCEs of the devices suggests that a thermally activated process may underlie the observed phenomenon. It is believed that the annealing process may facilitate the diffusion of O_2_ into the spiro-MeOTAD for inducing p-doping of the hole transport material. Furthermore, the process can result in lowering the localized state density at the grain boundaries as well as the bulk of perovskite. Utilizing thermal assisted O_2_ annealing, high efficiency devices with good reproducibility were attained. A PCE of 15.4% with an open circuit voltage (*V_OC_*) 1.04 V, short circuit current density (*J_SC_*) 23 mA/cm^2^, and fill factor 0.64 had been achieved for our champion device.

The dramatic emergence of the hybrid inorganic-organic perovskites as a photovoltaic material resulted in a remarkable impact on the field of photovoltaics^1^,^2^,^3^. Such impressive progress in the development of the perovskite-based photovoltaic cells is attributed to the desirable physical properties for this class of materials such as their broadly tunable bandgaps^4^, high absorption coefficients over a wide range of visible light spectrum^5^, extremely long carrier diffusion lengths^6^,^7^, good crystallinity^8^,^9^ and high carrier mobilities^9^,^10^,^11^, which have fulfilled most of the criteria required for manufacturing high efficiency solar cells. Starting from the first attempt of using perovskite as the sensitizer^12^ for solar cells in 2009, the PCEs of perovskite-based devices have been enhanced from 3.8%^12^ to around 15%^13^,^14^, which represents the most rapid rate of increase in recent years compared to other competing photovoltaic technologies. During the time of the composition of this paper, the PCEs of perovskite solar cells have been further boosted to 16.2% (certified) and 19.3% by the efforts of Jeon *et al*.^15^ and Zhou et al.^16^ With continuing development of the perovskite-based solar cells, the device efficiency which is comparable to the commercial single-crystalline silicon solar cells potentially can be achieved in the near future.

CH_3_NH_3_PbI_3_, with a bandgap of 1.5 eV, is the most studied perovskite-based photovoltaic material, which has been incorporated into different structures of electron injecting layers such as the compact titanium(IV) oxide (TiO_2_) layer^17^ and mesoporous TiO_2 _layer^14^,^18^,^19^,^20^,^21^ or combined with one-dimensional nanostructures such as TiO_2_ nanorods^22^, nanofibers^23^ and ZnO nanorods^24^,^25^,^26^ for the formation of high efficiency photovoltaic cells. Due to the fact that the architecture of the perovskite solar cells evolved from mesoscopic dye-sensitized solar cells (DSSCs), tremendous research efforts of employing perovskite absorbers have been initially focused on TiO_2_ mesostructures yielding PCEs as high as 15% for CH_3_NH_3_PbI_3_ based perovskite devices^14^. However, as demonstrated by M. M. Lee *et al*.^8^ and J. M. Ball *et al*.,^27^ high PCE can also be achieved even if the mesoporous TiO_2_ is replaced with an insulating alumina (Al_2_O_3_) mesoscopic scaffold. Due to the higher conduction band of Al_2_O_3_ compared to the lowest unoccupied molecular orbital (LUMO) of the perovskite layer, it is not possible for the electrons to be injected from the perovskite layer into the Al_2_O_3_ scaffold indicating that the photoexcited electrons are transported through the perovskite layer^8^. This discovery raises the question of whether the TiO_2_ mesostructure is necessary to achieve high efficiency perovskite solar cells. Recently, several research groups have demonstrated efficient perovskite solar cells in a planar structure without the mesoscopic scaffold^13^,^17^,^28^,^29^,^30^,^31^,^32^,^33^,^34^. This type of architecture allows for much simpler fabrication process, minimizes the chance of charge recombination at the interfaces and can be compatible with multijunction in hybrid tandem solar cells.

Among the published works, mixed halide perovskites^13^,^32^,^33^,^34^ (e.g. CH_3_NH_3_PbI_3-x_Cl_x_) or CH_3_NH_3_PbI_3_^17^ are commonly employed in the planar structure with compact TiO_2_ as the hole blocking layer, yielding the highest PCE of 15.4% for vapor deposited CH_3_NH_3_PbI_3-x_Cl_x_-based device^13^ and 12.1% for CH_3_NH_3_PbI_3_-based device prepared by vapor-assisted solution process^17^. Although a number of reports have demonstrated significant potential in obtaining high efficiency perovskite-based solar cells, the group-to-group, or even batch-to-batch, variations in the reported PCEs is substantial which is believed to arise from the high sensitivity to the fabrication techniques and ambient during the formation of the active layers of the devices. Further development in the field of perovskite-based photovoltaics demands the establishment of a series of optimized and controllable fabrication procedures for yielding highly efficient and reproducible devices.

In this work, we have investigated planar heterojunction devices with a structure that consists of glass/fluorine-doped tin oxide (FTO)/TiO_2_ compact layer/CH_3_NH_3_PbI_3_/2,2′7,7′-tetrakis(N,N-di-p-methoxyphenyl-amine)-9,9′-spirobifluorene (spiro-MeOTAD)/molybdenum(VI) oxide (MoO_3_)/aluminum (Al) as illustrated in Fig. 1a. The perovskite films are prepared by sequential two- step solution processed deposition method^14^. Two post-deposition treatments were investigated. The devices were annealed in dry O_2_ for a period of 12 hours at 4 different temperatures: room temperature (ART); 45°C (A45); 65°C (A65); and 85°C (A85). An annealing time of 12 hours was chosen to ensure that the oxidation process is complete. The annealing time can be further optimized and has to be shortened to enhance the cost-effectiveness of the process. The control samples (NRT) were stored in N_2_ at room temperature for 12 hours before the deposition of metal electrodes. It is observed that the performances of perovskite-based devices are strongly affected by the post-deposition treatments of the films. Through careful control of the fabrication processes, a highest PCE of 15.4% was achieved. The processing conditions, post-deposition treatments and corresponding results will be discussed in detail, which are essential for the future development of perovskite-based solar cells.

## Results

**Figure 1:**
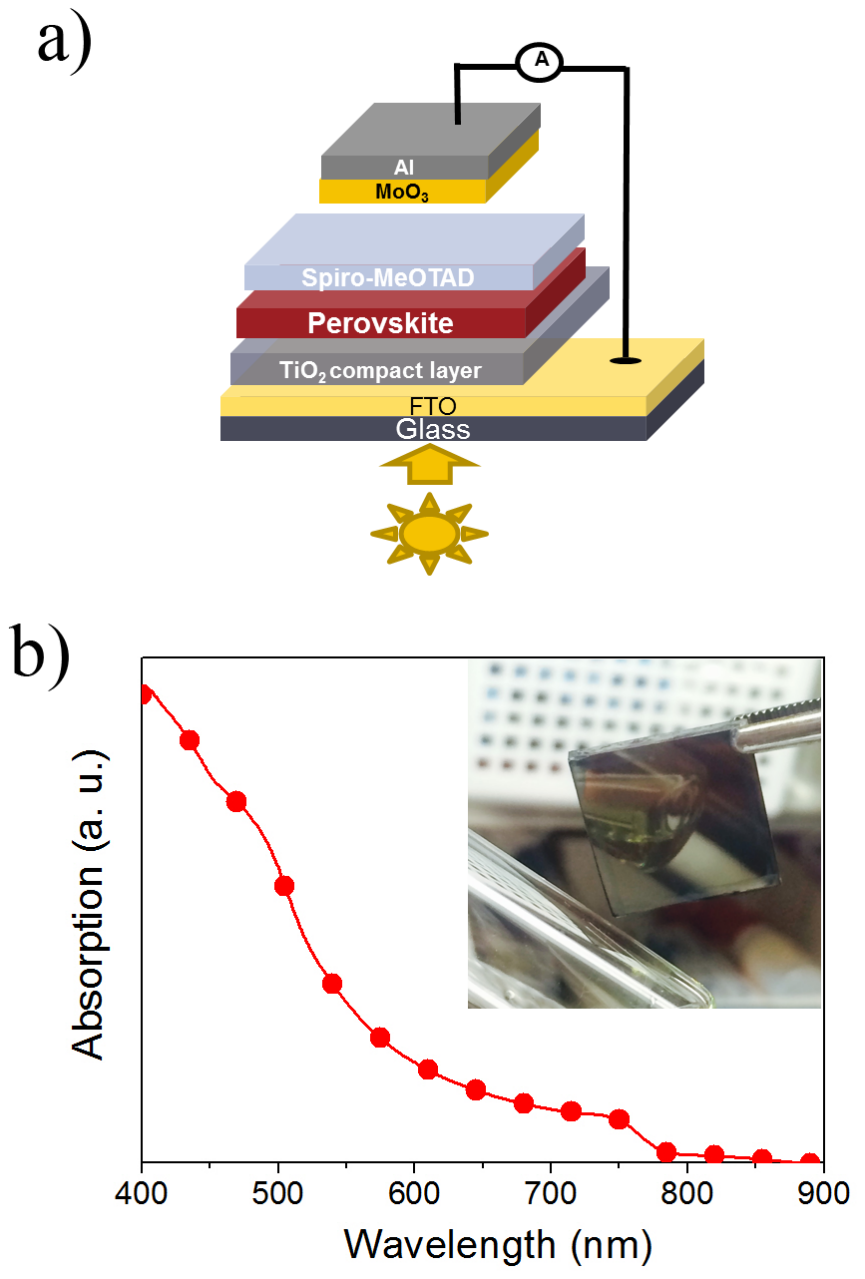
(a) The device architecture of CH_3_NH_3_PbI_3_ based solar cell (b) absorption spectra of CH_3_NH_3_PbI_3_ before and after O_2_ post-deposition treatments.

**Figure 2:**
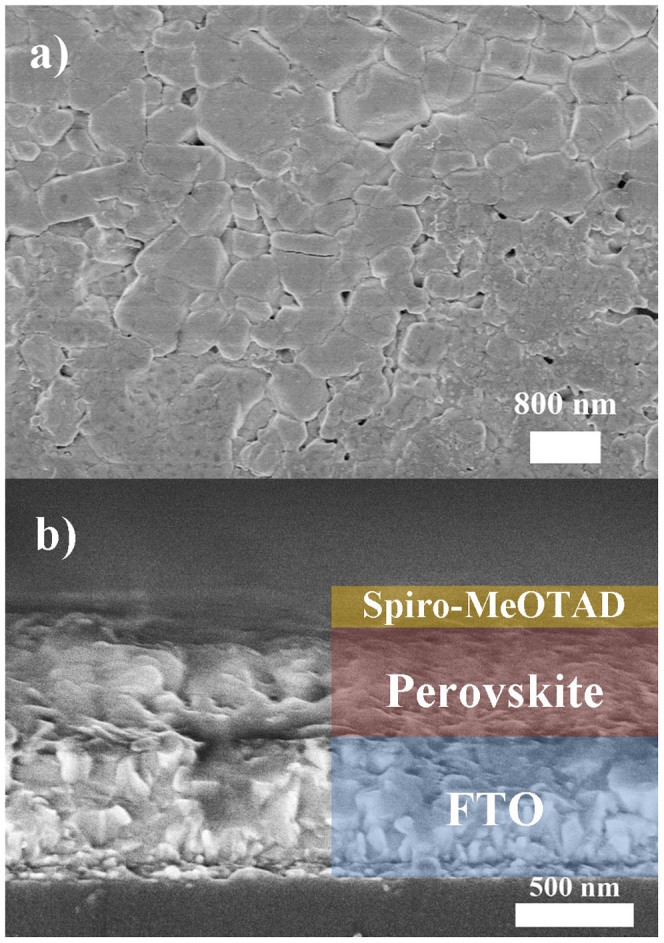
(a) Top-view and (b) cross-sectional SEM images for the sample of FTO/TiO_2_/CH_3_NH_3_PbI_3_/spiro-MeOTAD.

**Figure 3:**
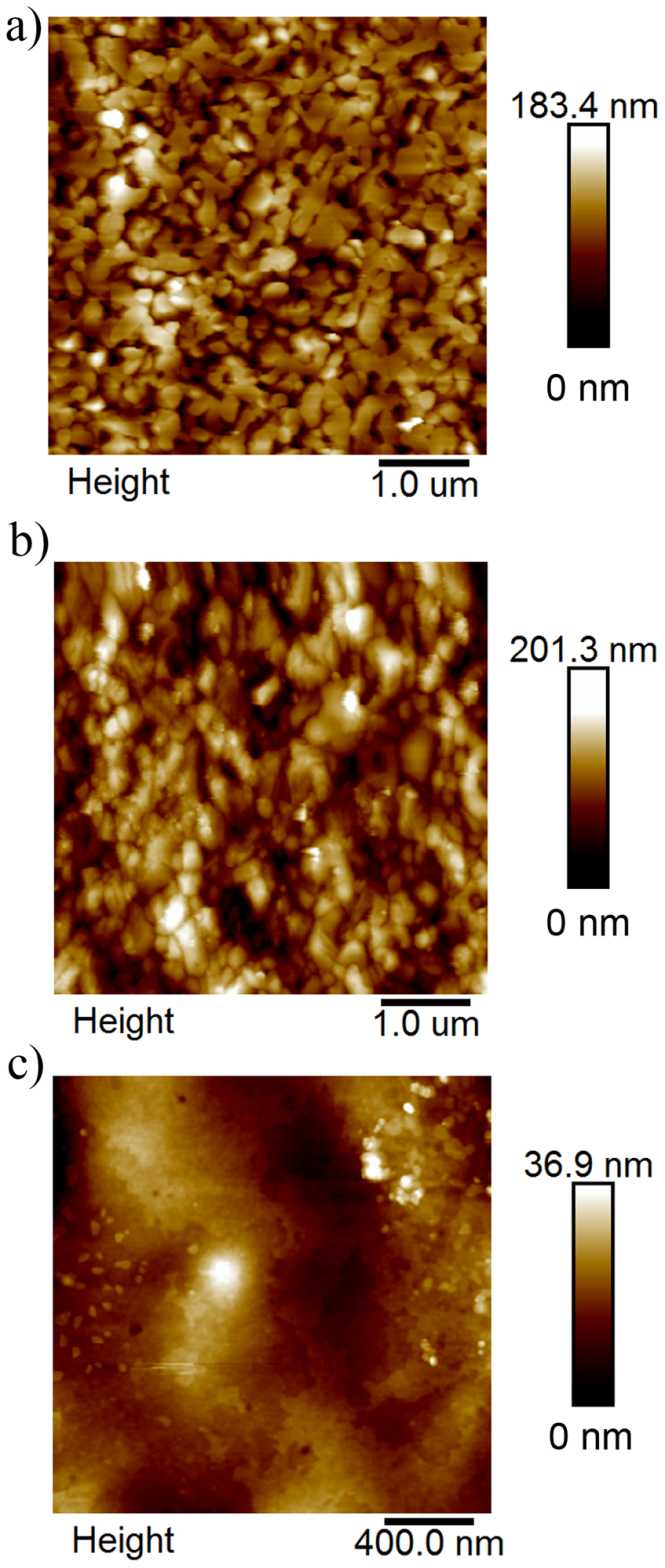
AFM images for (a) PbI_2_ (RMS: 25 nm) (b) CH_3_NH_3_PbI_3_ (RMS: 31 nm) and (c) CH_3_NH_3_PbI_3_/spiro-MeOTAD (RMS: 5 nm).

**Figure 4:**
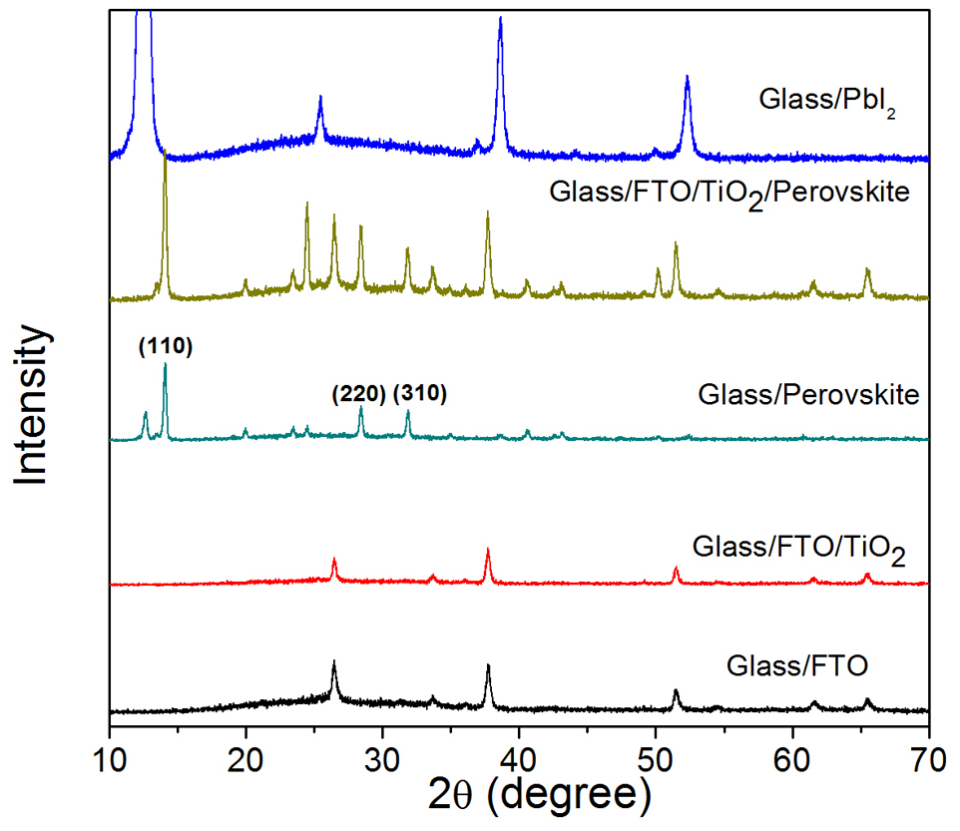
XRD patterns for the sample with different layers on glass or FTO coated glass.

CH_3_NH_3_PbI_3_-based solar cells have been fabricated and the device configuration is illustrated in Fig. 1a. The broad absorption spectrum of CH_3_NH_3_PbI_3_ is shown in Fig. 1b, indicating the strong photon harvesting capability of the material over the spectral range from 400 nm to 800 nm, which is in agreement with the band gap of 1.5 eV for CH_3_NH_3_PbI_3_^18^,^35^. The inset of Fig. 1b is a photograph of a CH_3_NH_3_PbI_3_ film on the FTO coated glass substrate. The prepared film with smooth and reflective surface can be clearly observed by naked eyes, which is believed to be a desirable quality for the development of high performance devices^31^. The morphology and cross section of the samples have been investigated by scanning electron microscopy (SEM) and the images of typical films (A65) are presented in Fig. 2. From the plan-view image of CH_3_NH_3_PbI_3_ formed on TiO_2_ compact layer, crystalline domains of perovskite with grain size in the range of ~100 nm to ~800 nm with good surface coverage is observed. High surface coverage is of great importance for high performance devices as undesired shunting paths are minimized and thereby maximizing photo-carrier collection^34^. Furthermore, crystals with large grain size are shown to be favorable for charge transport^17^,^36^ and may serve as light scattering centers^29^ and are, therefore, preferable for the development of high performance devices. The cross-sectional SEM image of a typical device without the top electrode is shown in Fig. 2b. The thicknesses of the solution-processed CH_3_NH_3_PbI_3_ and spiro-MeOTAD layers are around 410 nm and 190 nm respectively, which are in the range of the reported values for achieving high efficiency devices^8^,^14^,^19^. The surface morphologies of PbI_2_ and CH_3_NH_3_PbI_3_ with or without the spiro-MeOTAD layer on top have been characterized by atomic force microscopy (AFM) and the results are shown in Fig. 3. It is found that the root mean square (RMS) roughness significantly increases from 25 nm to 31 nm after the PbI_2_ and the CH_3_NH_3_I layers were allowed to react to form CH_3_NH_3_PbI_3_. An increase in the grain size is observed subsequent to the formation of CH_3_NH_3_PbI_3_, which is attributed to the volume expansion^17^,^37^ commonly observed in the process. The surface roughness of CH_3_NH_3_PbI_3_ with the hole transport layer (HTL, spiro-MeOTAD) on top is reduced to 5 nm, indicating that the perovskite layer is fully covered and hence short-circuit currents due to the direct contact between the relatively conductive perovskite (~10^−3^ S cm^−1^) and metal electrode can be avoided^8^. The samples of CH_3_NH_3_PbI_3_ deposited on glass or compact TiO_2_ coated FTO substrates were further characterized by X-ray diffraction (XRD) and the diffraction patterns are shown in Fig. 4. Strong diffraction peaks located at 14.0°, 28.5° and 31.8° for 2*θ* scan were observed corresponding to the planes of (110), (220) and (310), which are in good agreement with the previous reports^17^,^14^,^38^, indicating that the tetragonal perovskite structure is formed^5^,^12^,^37^.

**Figure 5:**
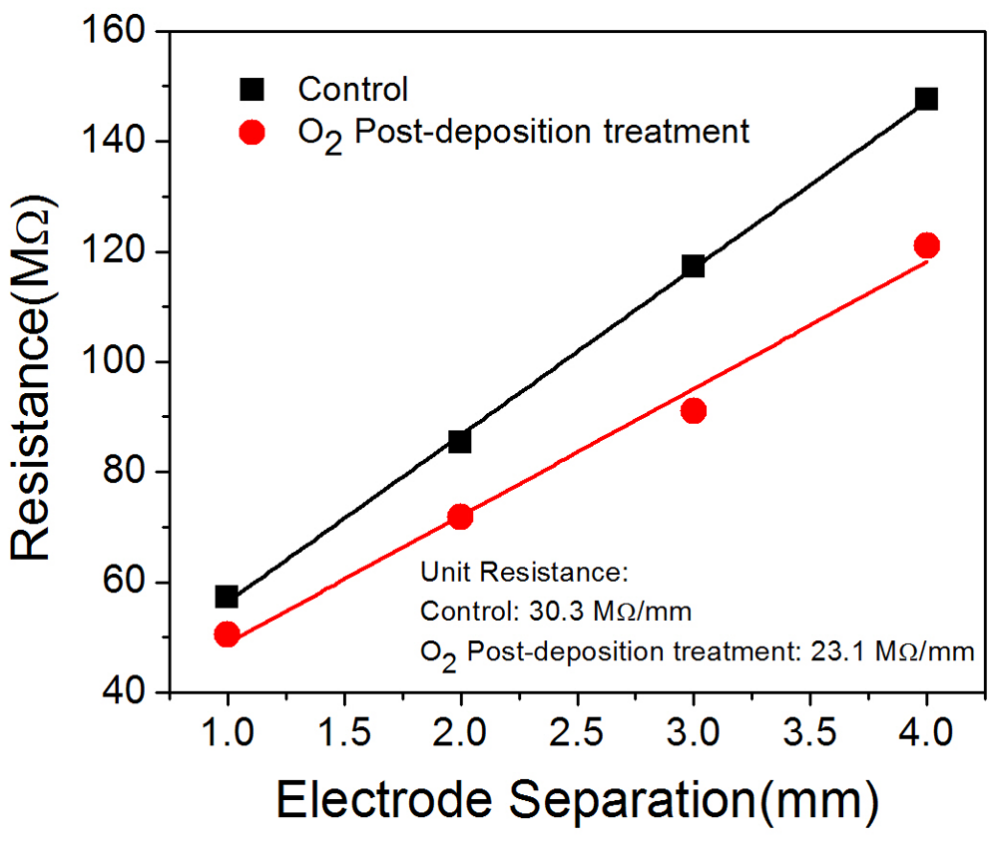
The resistance of the film of spiro-MeOTAD across the electrodes with and without O_2_ post-deposition treatments.

The CH_3_NH_3_PbI_3_-based planar devices were fabricated in an N_2_-filled glove box (O_2_ and H_2_O <0.1 ppm) since the perovskite materials are easily decomposed in the presence of moisture^39^. However, we found that the devices exhibit poor performance when the entire fabrication processes were carried out in the glove box. This is attributed to the poor doping conditions for the spiro-MeOTAD layer as it was pointed out that O_2_ may be essential for the doping mechanism of spiro-MeOTAD^40^,^41^,^42^. We have performed *I-V* measurements on the spiro-MeOTAD film using the Transfer Length Method (TLM). Resistances across the electrodes with different separations are shown in Fig. 5. The results clearly indicate significant reduction in the resistance of the spiro-MeOTAD layer annealed in O_2_ ambient. This is indicative of improved doping level for the annealed film. Similar observations of reduction in the cell efficiency for solid-state dye-sensitized solar cells fabricated in N_2_ had also been reported previously^42^. This situation differs from the fabrication of organic devices, such as organic light emitting devices and organic solar cells, for which exposure to O_2_ should be avoided as it can lead to severe device degradation^43^,^44^. Considering the fact that moisture is highly devastating to the performance of perovskite-based devices while O_2_ is necessary for obtaining high efficiency devices, we deposited all the different layers of the device in N_2_-filled glove box to prevent the exposure to moisture, after that the prepared samples were annealed in dry O_2_ (high purity grade, >99.9%) for 12 hours at different annealing temperatures.

**Figure 6:**
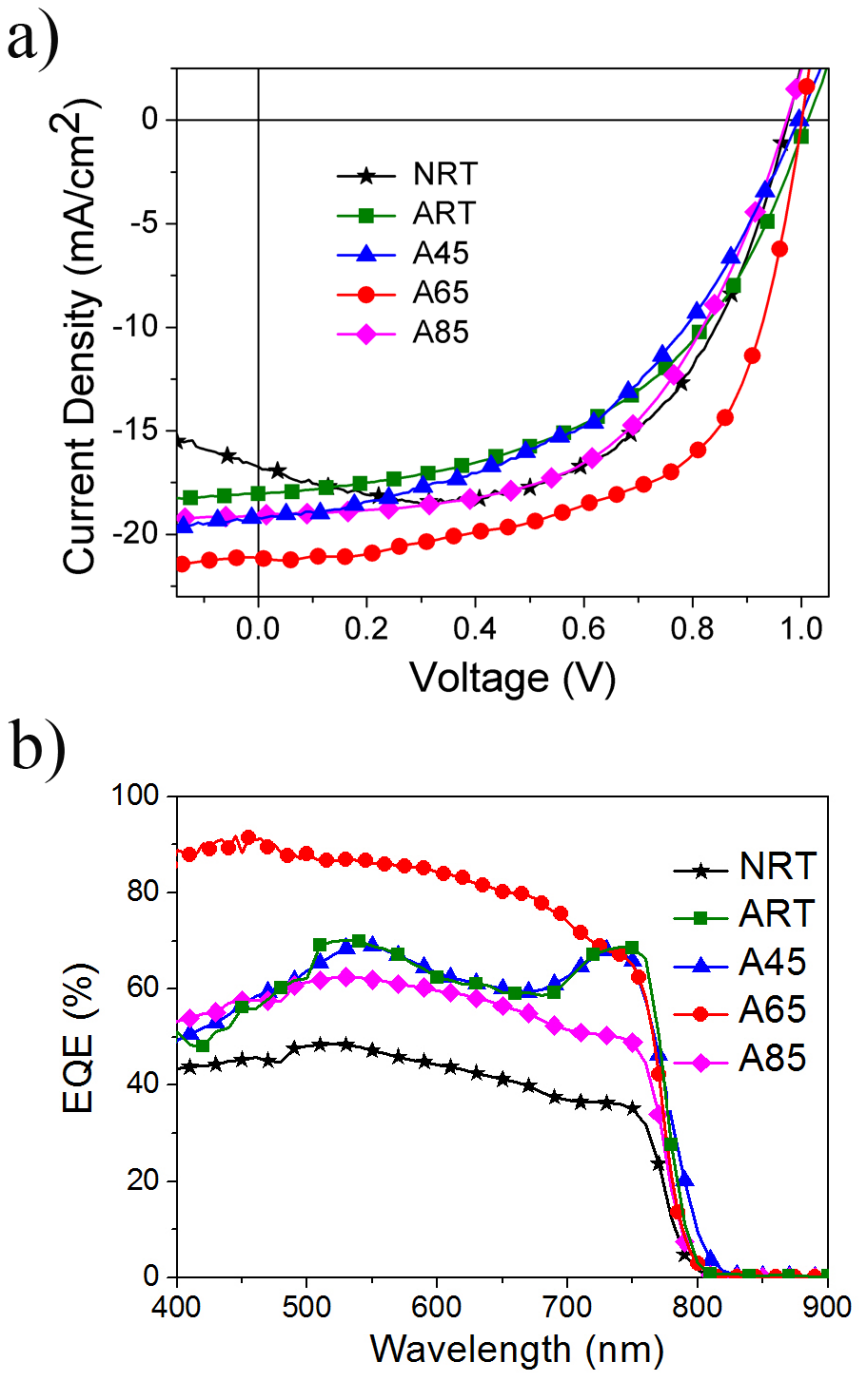
(a) The *I-V* curves and (b) EQE for representative devices with different post-deposition treatments.

The photovoltaic parameters of CH_3_NH_3_PbI_3_-based devices with different post-deposition treatments are summarized in Table 1 while the *I-V* curves of the representative devices, which are close to the average performance for each post-deposition condition, are presented in Fig. 6a. The experimental data demonstrates substantial enhancement in the PCE (from 6.0% to 8.5%) for the ART-devices compared to the control devices (NRT-devices). The device performances were further improved when the samples were annealed in O_2_ at an elevated temperature. Under the optimized temperature (65°C), the *J_SC_* and fill factor of the A65-devices are significantly enhanced, yielding an average PCE of 12.0%. Since there is no obvious change in surface morphology and absorption as observed from SEM (Fig. S1) and absorption measurement (Fig. 1b) before and after O_2_ annealing process, the improvement in device performance is believed to originate from the lowering of the defect densities of the perovskite films. It is believed that the thermal annealing process may facilitate the diffusion of O_2_ to the perovskite film as well as the material interfaces to passivate the under-coordinated cations of the perovskite layer leading to the reduction in the density of the trap states and thereby reducing the recombination rate, which is reflected by the trend of obtained *J_SC_* shown in Fig. 6a. It is found that the *J_SC_* of the devices strongly depends on the post-deposition treatments, which is observed to increase from an average of 11.7 mA/cm^2^ for NRT-devices to 21.0 mA/cm^2^ for A65-devices, suggesting that charges can be extracted more efficiently, likely due to the inhibition of charge recombination at the trap states. Similar work for passivation of halide anions resulting in improved device performance has also been reported previously^45^. However, further increasing the thermal annealing temperature to 85°C resulted in a drop in the *J_SC_*, fill factor and accordingly PCE, which is possibly due to thermally induced degradation of the materials. Besides, a decrease in the magnitude of the *J_SC_* for the NRT-devices for *V* < 0.3 V is observed, which could be attributed to the low conductivity of spiro-MeOTAD and high density of interface states in the absence of O_2_ annealing, resulting in poor charge collection efficiency. It should be noted that hysteresis effect is generally observed in the *I-V* characteristics of all our devices, which basically remains unchanged even after the O_2_ annealing process. This suggests that the O_2_ annealing process is not able to eliminate the processes/defects responsible for the observed hysteresis effect. The *I-V* curves shown in the figures were obtained from the reverse scan at the scan rate of 0.01 V/s from 1.2 V to −0.2 V.

The EQE spectra of CH_3_NH_3_PbI_3_-based devices with different post-deposition treatments are shown in Fig. 6b, which demonstrate wide spectral response from 400 nm to 800 nm in good agreement with the absorption spectra shown in Fig. 1b. The trend of the EQE spectra is also consistent with the *I-V* performance of the devices. There is a significant enhancement in EQE for the ART and A45 devices compared to the NRT devices. The EQE is further enhanced for the samples annealed in dry O_2_ at 65 °C while further increasing the annealing temperature to 85°C in the presence of O_2_ results in a reduction in the EQE. It is interesting to point out that a drop in the EQE results in the short wavelength range is typically attributed to high concentration of defect states in the front heterojunction. Detailed comparison between the EQE data for the control device (NRT) and the annealed device under optimal conditions (A65) we observe significant improvement in the EQE results at the short wavelength range. In general, the reduction in the EQE of solar cells can be attributed to charge recombination and short charge carrier diffusion length^46^, unequal enhancement in EQE along the spectrum is likely to occur when the condition of post-deposition treatment has not been completely optimized and considerable amount of traps still exist. It appears that O_2_ annealing may lead to an improvement in the carrier collection efficiency across the TiO_2_/perovskite interface^47^and the grain boundaries within the perovskite layer.

**Figure 7:**
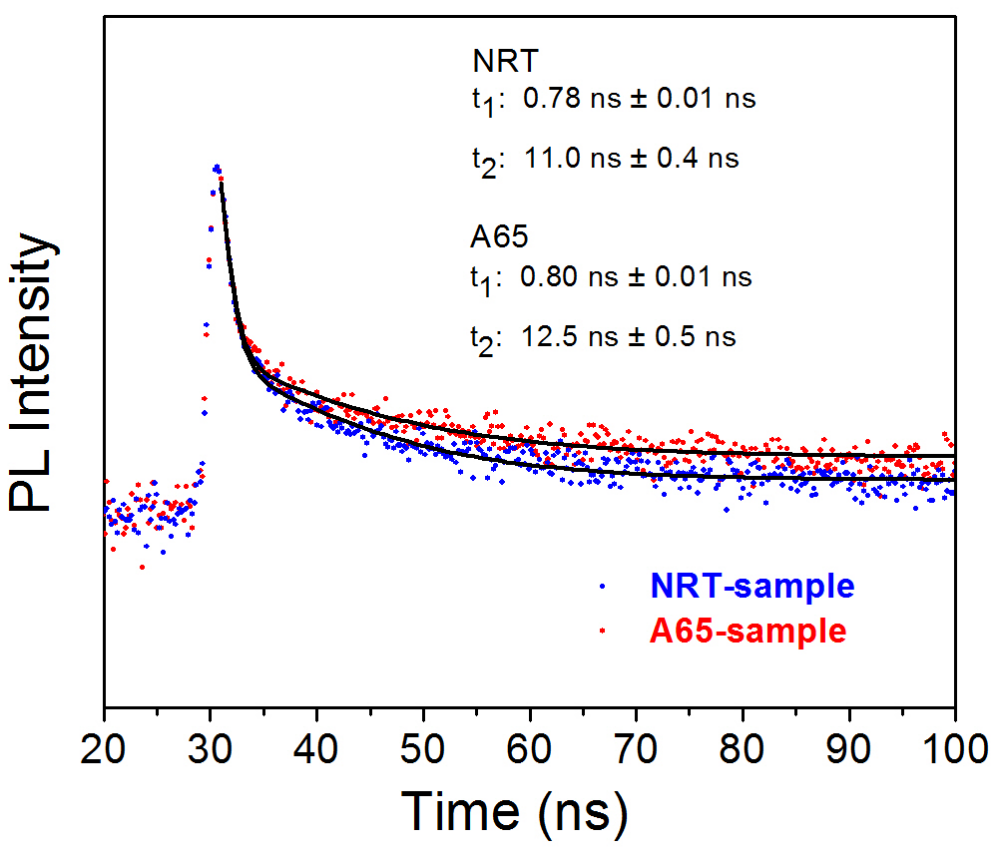
The time-resolved photoluminescence of the bare perovskite film with or without O_2_ post-deposition treatment.

Detailed characterization of the time-resolved photoluminescence signal has been performed for both the control sample and the perovskite film annealed in O_2_ ambient at 65°C. The films were deposited directly on quartz substrates in order to pinpoint the effects of the O_2_ annealing process on the perovskite film. The experimental data are shown in Fig. 7. It is clear from the data that two separate lifetimes exist in the annihilation of the PL signal indicative of the presence of two recombination pathways^48^. Substantial improvements in the carrier lifetimes are observed in the O_2_ annealed sample indicating significant reduction in the density of the localized states in the perovskite film, which is consistent with the results of device performance. It is likely that most of these localized states reside at the grain boundaries however based on the data alone it is not possible to identify the specific location of the trap states. Further work needs to be done to specifically identify the mechanism underlying the observed phenomenon.

**Figure 8:**
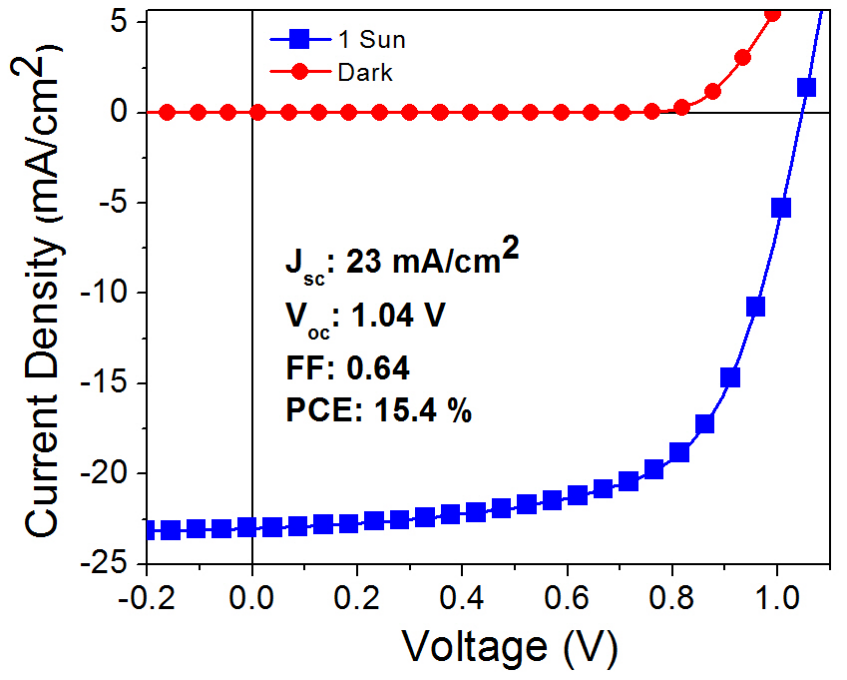
(a) The *I-V* curve of the best device measured at 100 mW/cm^2^ and in dark.

By adopting the strategy of thermally assisted O_2_ annealing of the deposited films, a champion device with a PCE as high as 15.4% and a high *J_SC_* of 23 mA/cm^2^ can be achieved and the corresponding *I-V* curves are shown in Fig. 8. It is noted that the reproducibility of the perovskite-based solar cells can be significantly improved by post-deposition treatments, which is reflected by the lower values in the standard deviations in the photovoltaic parameters indicated in Table 1. The proposed fabrication strategy is straightforward and controllable for assembling highly efficient and good reproducible perovskite-based solar cells.

## Discussion

CH_3_NH_3_PbI_3_-based devices have been fabricated under well controlled fabrication procedures. Our work demonstrated that post-deposition O_2_ treatments are critical, which not only enhance the device performance substantially, but also improve the device reproducibility. We found that placing the solution-processed films in dry O_2_ at 65°C prior to electrode deposition is an essential process for device fabrication, yielding an averaged enhanced PCE of 12%. Our results suggest that the improvement in device performance by O_2_ annealing process is due to the enhancement in the conductivity of spiro-MeOTAD as well as the reduction in the defect density of the perovskite film.

## Methods

### Materials

Patterned FTO coated glass substrates with a sheet resistance of 7-10 Ω/□ were supplied from KINTEC company. Lead (II) iodide (purity 99%, PbI_2_), titanium (IV) isopropoxide (TTIP), lithium bis(trifluoromethylsulphonyl)imide (Li-TFSI), 4-tert-butylpyridine (tBP), N,N-dimethylformamide (DMF) and MoO_3_ were purchased from Sigma-Aldrich while spiro-MeOTAD (purity> 99.5%) was purchased from Luminescence Technology Corp. Methylammonium iodide (CH_3_NH_3_I) was purchased from Dyenamo. All chemicals were used as received.

### Device fabrication

Patterned FTO on glass substrates were cleaned sequentially by ultrasonication in toluene, acetone, ethanol and deionized water. The substrates were dried by the nitrogen flow and then exposed to UV-ozone for 20 min prior to the spin coating step. TiO_2_ compact layer was prepared by spin coating a mildly acidic diluted TTIP solution (1.25 ml) in ethanol (25 ml) at 3000 rpm followed by sintering at 450°C for 2 hours. CH_3_NH_3_PbI_3_ was synthesized by a two-step technique^14^ with optimized solution concentrations in N_2_ filled glove box. PbI_2_ films were prepared by spin coating a solution of PbI_2_ (1500 rpm) dissolved in DMF with a concentration of 462 mg/ml which were then annealed on a hotplate at 70°C for 1 hour prior to the reaction with CH_3_NH_3_I (30 mg/ml in isopropanol). The samples were then annealed at 90°C to ensure complete reaction between PbI_2_ and CH_3_NH_3_I. The hole transport layer (HTL) was prepared by spin coating a solution of spiro-MeOTAD (80 mg/ml) dissolved in chlorobenzene with the additives of Li-TFSI (17.5 µL from a stock solution of 520 mg/ml in acetonitrile) and 29 µL of tBP at 4500 rpm. The prepared samples were then stored in N_2_ ambient at room temperature or in O_2_ (high purity grade >99.9%) ambient at a flow rate of 2 lit/min at room temperature, 45°C, 65°C or 85°C for 12 hours. Electrodes (MoO_3_ (15 nm)/Al (120 nm)) were then deposited by thermal evaporation through a shadow mask and the device area was 0.1 cm^2^.

### Device and sample characterizations

The *I-V* characteristics of the devices were measured using a B1500 A semiconductor parameter analyzer under the calibrated ABET Technologies SUN 2000 solar simulator equipped with an AM 1.5 filter at 100 mW/cm^2^. The *I-V* curves are obtained from the reverse scan at the scan rate of 0.01 Vs^−1^ from 1.2 V to −0.2 V. External quantum efficiency (EQE) was determined by a QE system from Enli Technology Co. Ltd. The surface morphologies of the films were characterized by atomic force microscopy (AFM) in the tapping mode using a Bruker NanoScope 8. UV-visible spectroscopy was performed by using a UV-2550 Shimadzu UV-VIS spectrophotometer for the perovskite film deposited on quartz. X-ray diffraction (XRD) patterns was determined by using a Rigaku SmartLab X-ray diffractometer in a step of 0.01° for 2θ from 10° to 70°. Scanning electron microscopy (SEM) was performed by using Hitachi S-4800 field emission scanning electron microscope. Time-resolved photoluminescence signals of perovskite film were monitored at 775 nm and recorded by using Edinburgh FLSP920 spectrophotometer equipped with the excitation source of 485 nm picosecond pulsed diode laser.

## Author Contributions

C.S. proposed and supervised the whole project. Z.R. contributed to the device fabrication and process optimization for the entire project. A.B.D., W.W.-F.L. and W.K.C. involved the discussion of the experiments. Q.S. and A.N. assisted the preparation of experiments. J.W. and L.Y. prepared the solution of TiO_2_. Q.S. carried out the XRD and spiro-MeOTAD resistance measurement. H.C.G. performed the characterization of prepared films by AFM. Y.W.-K. examined the film morphology by SEM. J.H. and G.B. conducted the measurement of time-resolved photoluminescence. A.N. carried out the EQE measurement and wrote the manuscript.

## Supplementary Material

Supplementary Information Thermal Assisted Oxygen Annealing for High Efficiency Planar CH_3_NH_3_PbI_3_ Perovskite Solar Cells

## Figures and Tables

**Table 1:** The average values of photovoltaic parameters obtained from I-V measurements for planar CH_3_NH_3_PbI_3_ based solar cells with different post-deposition treatments and integrated photocurrent from the EQE spectra are indicated in the bracket

Type	*V_OC_* (V)	*J_SC_* (mA/cm^2^)	FF	η (%)	*Δ*η (%)
NRT	0.96 ± 0.02	11.7 ± 4.0 (10.6)	0.48 ± 0.20	6.0 ± 3.7	-
ART	0.90 ± 0.12	19.5 ± 1.2 (15.3)	0.48 ± 0.09	8.5 ± 2.6	42
A45	0.99 ± 0.03	19.3 ± 0.4 (15.6)	0.48 ± 0.03	9.2 ± 0.9	53
A65	0.98 ± 0.01	21.0 ± 0.5 (20.5)	0.57 ± 0.03	12.0 ± 0.7	100
A85	0.97 ± 0.02	17.9± 2.6 (14.0)	0.49 ± 0.02	8.6 ± 1.0	43

## References

[b1] Kim H.-S., Im S. H. & Park N.-G. Organolead halide perovskite: New horizons in solar cell research. J. Phys. Chem. C 118, 5615–5625 (2014).

[b2] Boix P. P., Nonomura K., Mathews N. & Mhaisalkar S. G. Current progress and future perspectives for organic/inorganic perovskite solar cells. Mater. Today 17, 16–23 (2014).

[b3] Snaith H. J. Perovskites: The emergence of a new era for low-cost, high efficiency solar cells. J. Phys. Chem. Lett. 4, 3623–3630 (2013).

[b4] Eperon G. E. *et al.* Formamidinium lead trihalide: a broadly tunable perovskite for efficient planar heterojunction solar cells. Energy Environ. Sci. 7, 982–988 (2014).

[b5] Im J.-H., Lee C.-R., Lee J.-W., Park S.-W. & Park N.-G. 6.5% efficient perovskite quantum-dot-sensitized solar cell. Nanoscale 3, 4088–4093 (2011). 21897986 10.1039/c1nr10867k

[b6] Stranks S. D. *et al.* Electron-hole diffusion lengths exceeding 1 micrometer in an organometal trihalide perovskite absorber. Science 342, 341–344 (2013). 24136964 10.1126/science.1243982

[b7] Xing G. *et al.* Long-range balanced electron- and hole-transport lengths in organic-inorganic CH_3_NH_3_PbI_3_. Science 342, 344–347 (2013). 24136965 10.1126/science.1243167

[b8] Lee M. M., Teuscher J., Miyasaka T., Murakami T. N. & Snaith H. J. Efficient hybrid solar cells based on meso-superstructured organometal halide perovskites. Science 338, 643–647 (2012) 23042296 10.1126/science.1228604

[b9] Stoumpos C. C., Malliakas C. D. & Kanatzidis M. G. Semiconducting tin and lead iodide perovskites with organic cations: Phase transitions, high mobilities, and near-infrared photoluminescent properties. Inorg. Chem. 52, 9019–9038 (2013). 23834108 10.1021/ic401215x

[b10] Hodes G. Perovskite-based solar cells. Science 342, 317–318 (2013). 24136955 10.1126/science.1245473

[b11] Mitzi D. B. Synthesis, Structure, and Properties of Organic-Inorganic Perovskites and Related Materials. Prog. Inorg. Chem. 48, 1–121 (2007).

[b12] Kojima A., Teshima K., Shirai Y. & Miyasaka T. Organometal halide perovskites as visible-light sensitizers for photovoltaics cells. J. Am. Chem. Soc. 131, 6050–6051 (2009). 19366264 10.1021/ja809598r

[b13] Liu M., Johnston M. B. & Snaith H. J. Efficient planar heterojunction perovskite solar cells by vapour deposition. Nature 501, 395–398 (2013). 24025775 10.1038/nature12509

[b14] Burschka J. *et al.* Sequential deposition as a route to high-performance perovskite-sensitized solar cells. Nature 499, 316–319 (2013). 23842493 10.1038/nature12340

[b15] Jeon N. J. *et al.* Solvent engineering for high-performance inorganic-organic hybrid perovskite solar cells. Nat. Mater. 13, 897–903 (2014). 24997740 10.1038/nmat4014

[b16] Zhou H. *et al.* Interface engineering of highly efficient perovskite solar cells. Science 345, 542–546 (2014). 25082698 10.1126/science.1254050

[b17] Chen Q. *et al.* Planar heterojunction perovskite solar cell via vapor-assisted solution process. J. Am. Chem. Soc. 136, 622–625 (2014). 24359486 10.1021/ja411509g

[b18] Kim H.-S. *et al.* Lead iodide perovskite sensitized all-solid-state submicron thin film mesoscopic solar cells with efficiency exceeding 9%. Sci. Rep. 2, 591; 10.1038/srep00591 (2012). 22912919 10.1038/srep00591 PMC3423636

[b19] Bi D. *et al.* Using a two-step deposition technique to prepare perovskite (CH_3_NH_3_PbI_3_) for thin film solar cells based on ZrO_2_ and TiO_2_ mesostructures. RCS Adv. 3, 18762–18766 (2013).

[b20] Heo J. H. *et al.* Efficient inorganic-organic hybrid heterojunction solar cells containing perovskite compound and polymeric hole conductor. Nat. Photonics 7, 486–491 (2013).

[b21] Christians J. A., Fung R. C. M. & Kamat P. V. An inorganic hole conductor for organo-lead halide perovskite solar cells. Improved hole conductivity with copper iodide. J. Am. Chem. Soc. 136, 758–764 (2014). 24350620 10.1021/ja411014k

[b22] Kim H.-S. *et al.* High efficiency solid-state sensitized solar cell-based on submicrometer rutile TiO_2_ nanorod and CH_3_NH_3_PbI_3_ perovskite sensitizer. Nano Lett. 13, 2412–2417 (2013). 23672481 10.1021/nl400286w

[b23] Dharani S. *et al.* High efficiency electrospun TiO_2_ nanofiber based hybrid organic-inorganic perovskite solar cell. Nanoscale 6, 1675–1679 (2014). 24336873 10.1039/c3nr04857h

[b24] Son D.-Y., Im J.-H., Kim H.-S. & Park N.-G. 11% Efficient perovskite solar cell based on ZnO nanorods: an effective charge collection system. J. Phys. Chem. C 118, 16567–16573 (2014).

[b25] Bi D. *et al.* Efficient and stable CH_3_NH_3_PbI_3_-sensitized ZnO nanorods array solid-state solar cells. Nanoscale 5, 11686–11691 (2013). 24100947 10.1039/c3nr01542d

[b26] Kumar M. H. *et al.* Flexible, low-temperature, solution processed ZnO-based perovskite solid state solar cells. Chem. Commun. 49, 11089–11091 (2013). 10.1039/c3cc46534a 24141601

[b27] Ball J. M., Lee M. M., Hey A. & Snaith H. J. Low-temperature processed meso-superstructured to thin-film perovskite solar cells. Energy Environ. Sci. 6, 1739–1743 (2013).

[b28] Malinkiewicz O. *et al.* Perovskite solar cells employing organic charge- transport layers. Nat. Photonics 8, 128–132 (2014).

[b29] Liu D. & Kelly T. L. Perovskite solar cells with a planar heterojunction structure prepared using room-temperature solution processing techniques. Nat. Photonics 8, 133–138 (2014).

[b30] Liang P.-W. *et al.* Additive enhanced crystallization of solution-processed perovskite for highly efficient planar-heterojunction solar cells. Adv. Mater. 26, 3748–3754 (2014). 24634141 10.1002/adma.201400231

[b31] Jeng J.-Y. *et al.* CH3NH3PbI3 perovskite/fullerene planar-heterojunction hybrid solar cells. Adv. Mater. 25, 3727–3732 (2013). 23775589 10.1002/adma.201301327

[b32] Saliba M. *et al.* Influence of thermal processing protocol upon the crystallization and photovoltaic performance of organic-inorganic lead trihalide perovskites. J. Phys. Chem. C 118, 17171–17177 (2014).

[b33] Conings B. *et al.* Perovskite-based hybrid solar cells exceeding 10% efficiency with high reproducibility using a thin films sandwich approach. Adv. Mater. 26, 2041–2046 (2014). 24338932 10.1002/adma.201304803

[b34] Eperon G. E., Burlakov V. M., Docampo P., Goriely A. & Snaith H. J. Morphological control for high performance solution-processed planar heterojunction perovskite solar cells. Adv. Funct. Mater. 24, 151–157 (2014).

[b35] Wolf S. D. *et al.* Organometallic halide perovskite: Sharp Optical Absorption Edge and its relation to photovoltaic performance. J. Phys. Chem. Lett. 5, 1035–1039 (2014) 10.1021/jz500279b 26270984

[b36] Mohammed I. M., Elbadawi A. A. & Abuellhassan H. H. Temperature and grain size effect on the electrical conductivity of La_0.67_Ca_0.33_MnO_3_. Journal of Applied and Industrial Sciences 1, 12–22 (2013).

[b37] Baikie T. *et al.* Synthesis and crystal chemistry of the hybrid perovskite (CH_3_NH_3_)PbI_3_ for solid-state sensitised solar cell applications. J. Mater. Chem. A 1, 5628–5641 (2013).

[b38] Sun S. *et al.* The origin of high efficiency in low-temperature solution-processable bilayer organometal halide hybrid solar cells. Energy Environ. Sci. 7, 399–407 (2014).

[b39] Noh J. H., Im S. H., Heo J. H., Mandal T. N. & Seok S. I. Chemical Management for colorful, efficient, and stable inorganic−organic hybrid nanostructured solar cells. Nano Lett. 13, 1764–1769 (2013). 23517331 10.1021/nl400349b

[b40] Abate A. *et al.* Lithium salts as “redox active” p-type dopants for organic semiconductor and their impact in solid-state dye-sensitized solar cells. Phys. Chem. Chem. Phys. 15, 2572–2579 (2013). 23310946 10.1039/c2cp44397j

[b41] Ono L. K. *et al.* Air-exposure-induced gas molecule incorporation into spiro-MeOTAD films. J. Phys. Chem. Lett. 5, 1374–1379 (2014). 10.1021/jz500414m 26269982

[b42] Cappel U. B., Daeneke T. & Bath U. Oxygen-induced doping of spiro-MeOTAD in solid-state dye-sensitized solar cells and its impact on device performance. Nano Lett. 12, 4925–4931 (2012). 10.1021/nl302509q 22913390

[b43] So F. & Kondakov D. Degradation mechanisms in small-molecule and polymer organic light-emitting diodes. Adv. Mater. 22, 3762–3777 (2010). 20491088 10.1002/adma.200902624

[b44] Hermenau M. *et al.* Water and oxygen induced degradation of small molecule organic solar cells. Sol. Energy Mater. *& * Sol. Cells 95, 1268–1277 (2011).

[b45] Abate A. *et al.* Supramolecular halogen bond passivation of organic-inorganic halide perovskite solar cells. Nano Lett. 14, 3247–3254 (2014). 24787646 10.1021/nl500627x

[b46] Barkhouse D. A. R., Pattantyus-Abraham A. G., Levina L. & Sargent E. H. Thiols passivate recombination centers in colloidal quantum dots leading to enhanced photovoltaic device efficiency. ACS Nano 2, 2356–2362 (2008). 19206403 10.1021/nn800471c

[b47] Lira-Cantu M., Norrman K., Andreasen J. W. & Krebs F. C. Oxygen release and exchange in niobium oxide MEHPPV hybrid solar cells. Chem. Mater. 18, 5684–5690 (2006).

[b48] Chen Q. *et al.* Controllable self-induced passivation of hybrid lead iodide perovskites toward high performance solar cells. Nano Lett. 14, 4158–4163 (2014). 24960309 10.1021/nl501838y

