# Supplementary material for: Thermal Assisted Oxygen Annealing for High Efficiency Planar CH3NH3PbI3 Perovskite Solar Cells
Source: Sci Rep. 2014 Oct 24;4:6752. doi: 10.1038/srep06752 (PMC4208060; doi:10.1038/srep06752)
Supplement: Supplementary Information — Thermal Assisted Oxygen Annealing for High Efficiency Planar CH3NH3PbI3 Perovskite Solar Cells [file srep06752-s1.pdf]

[Supporting information]

Thermal Assisted Oxygen Annealing for High Efficiency Planar $CH_3NH_3PbI_3$ Perovskite Solar Cells

Zhiwei Ren[1], Annie Ng[1], Qian Shen[1], Huseyin Cem Gokkaya[1], Jingchuan Wang[2], Lijun Yang[2], Wai-Kin Yiu[3], Gongxun Bai[4], Aleksandra B. Djurišić[3], Wallace Woon-fong Leung[2], Jianhua Hao[4], Wai Kin Chan[5] and Charles Surya[1]

[1]Department of Electronic and Information Engineering, The Hong Kong Polytechnic University, Hong Kong SAR

[2]Department of Mechanical Engineering, The Hong Kong Polytechnic University, Hong Kong SAR

[3]Department of Physics, The University of Hong Kong, Pokfulam, Hong Kong SAR

[4] Department of Applied Physics, The Hong Kong Polytechnic University, Hong Kong SAR

[5]Department of Chemistry, The University of Hong Kong, Pokfulam, Hong Kong SAR

Correspondence and requests for materials should be addressed to C.S. (charles.surya@polyu.edu.hk)

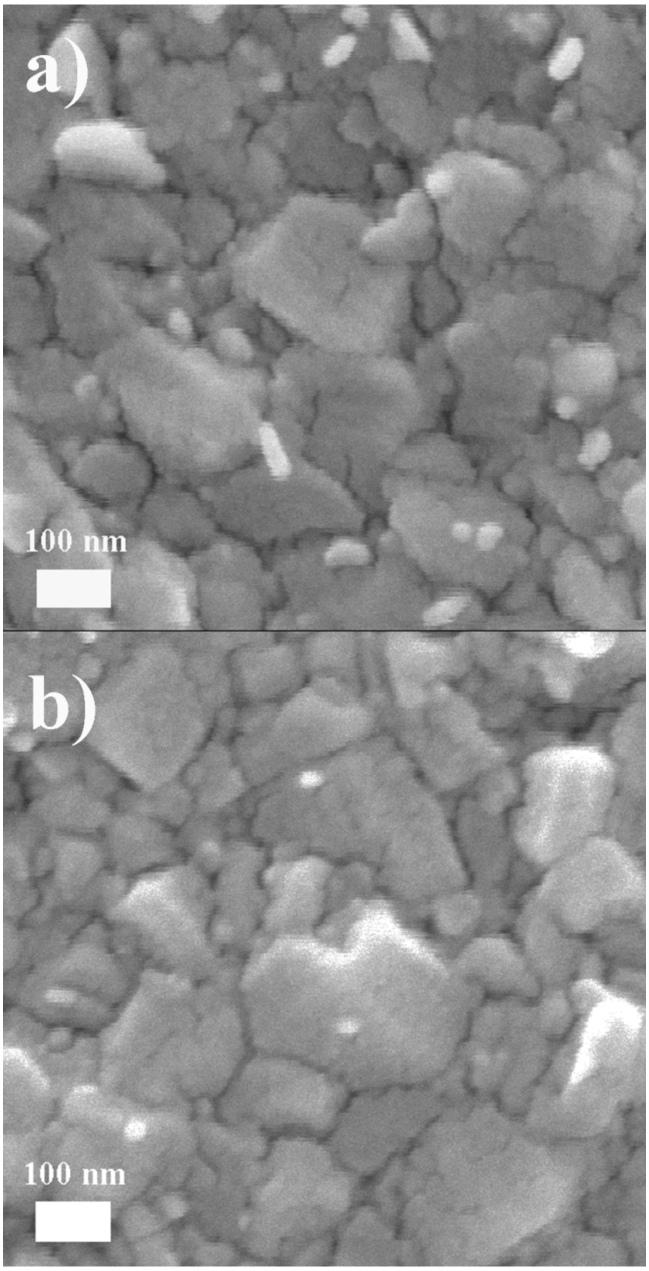

Figure S1. The SEM top-view image of the perovskite film before and after O_2 annealing process

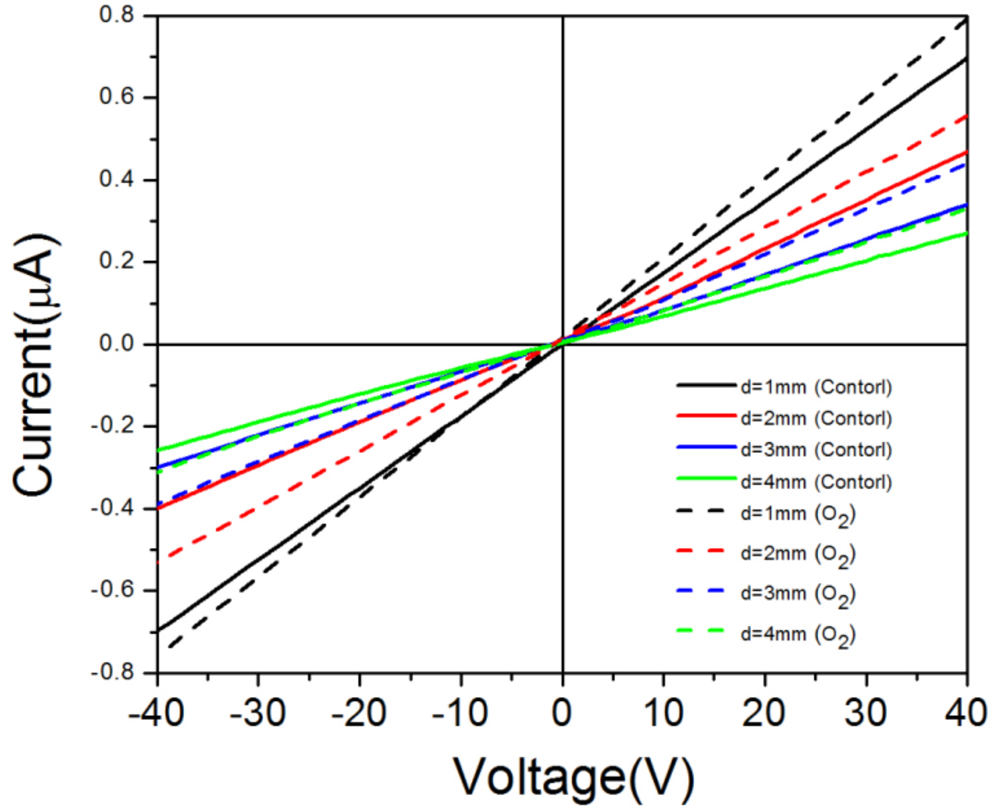

Figure S2. The *I-V* characteristics across the electrode with different separation on the film of spiro-MeOTAD before and after O_2 annealing
